# Arbuscular mycorrhizal fungi (AMF) enhanced the growth, yield, fiber quality and phosphorus regulation in upland cotton (*Gossypium hirsutum* L.)

**DOI:** 10.1038/s41598-020-59180-3

**Published:** 2020-02-07

**Authors:** Xinpeng Gao, Huihui Guo, Qiang Zhang, Haixia Guo, Li Zhang, Changyu Zhang, Zhongyuan Gou, Yan Liu, Junmei Wei, Aiyun Chen, Zhaohui Chu, Fanchang Zeng

**Affiliations:** 1 State Key Laboratory of Crop Biology, Shandong Agricultural University, Tai’an, 271018 P. R. China; 2 Novogene Bioinformatics Institute, Beijing, 100083 P. R. China

**Keywords:** Plant sciences, Plant physiology

## Abstract

We previously reported on the strong symbiosis of AMF species (*Rhizophagus irregularis* CD1) with the cotton (*Gossypium hirsutum* L.) which is grown worldwide. In current study, it was thus investigated in farmland to determine the biological control effect of AMF on phosphorus acquisition and related gene expression regulation, plant growth and development, and a series of agronomic traits associated with yield and fiber quality in cotton. When AMF and cotton were symbiotic, the expression of the specific phosphate transporter family genes and P concentration in the cotton biomass were significantly enhanced. The photosynthesis, growth, boll number per plant and the maturity of the fiber were increased through the symbiosis between cotton and AMF. Statistical analysis showed a highly significant increase in yield for inoculated plots compared with that from the non inoculated controls, with an increase percentage of 28.54%. These findings clearly demonstrate here the benefits of AMF-based inoculation on phosphorus acquisition, growth, seed cotton yield and fiber quality in cotton. Further improvement of these beneficial inoculants on crops will help increase farmers’ income all over the world both now and in the future.

## Introduction

Cotton is an important natural fiber and economic crop that provides substantial benefits to humans and is an important raw material worldwide. While a large number of recent studies have confirmed that arbuscular mycorrhizal fungi (AMF) can improve the growth, yield, quality, and phosphorus acquisition in plants^1–5^, the effect of AMF on these economic and agronomic traits in cotton is largely unknown. Previous research evidence suggests that mycorrhiza-mediated inoculants can reduce the need for P fertilization by at least 25% (and even up to 50%) without any decrease in crop yield^6^. We also investigated and reported recently as Zhang *et al*. on the field biological control effect on cotton *Verticillium* wilt of AMF application.

The nutrient acquisition of crops is mainly controlled by root and soil microorganisms^7^, which play a key role in nutrient circulation and absorption and in fighting against soil pathogens^8,9^. Beneficial soil microorganisms are an important part of agricultural ecosystems^7,10,11^ and have been used in agriculture more frequently over the past few decades. Nevertheless, the cultivation of the target microorganism is not a simple task, given the large number of microbes, their functional diversity, and the complexity of microbial assemblages. AMF are important beneficial soil microbiota which can be cultured in the laboratory and have established close symbioses with plants over the past 455 million years^12^.

Arbuscular mycorrhizal fungi (AMF) colonize plant roots and can improve the adaptability of host plants, especially by offering additional phosphorus (P)^13,14^, nitrogen (N), and zinc^15^ to plants. Root systems were extended, increasing the root surface that is utilized for nutrient uptake by more than 100-fold, by symbiosis with AMF^15^. A large group of agricultural crops such as wheat, rice, corn, potato, tomato, onion, pulses, cotton, and soybean can form symbiotic relationships with AMF. These crops are all important in the world. Some domesticated AMF are available worldwide, and their use in agricultural fields as mycorrhiza-based inoculants is increasing^16^. Bioreactors *in vitro* or conventional pot co culture *in vivo* are used to produce AMF inoculants. Furthermore, some specific mycorrhizal inoculants can be available on the farmland^17^. The influence level of AMF on crop yield and economics of farming is still uncertain, particularly in wheat^11,18,19^, But in potatoes, AMF have been determined to increase total crop yield by 9.5%^20^. AMF also increase plant drought tolerance^5^ and protect against some fungal pathogens^21^. In addition, AMF may function throughout the entire growth period^22^ and affect whole-plant physiological responses^4,23–25^^.^

The purpose of this study was to directly survey the effects of *Rhizophagus irregularis* CD1, the highest-efficiency fungus for symbiosis with the cotton cultivar Lumian No. 1, one of the best cultivated varieties in China, on cotton grown in the field under a reduced-fertilization program. We paid specific attention to the growth, seed cotton yield, fiber quality and phosphorus acquisition of the cotton (*Gossypium hirsutum* L.)

## Materials and Methods

### Arbuscular mycorrhizal fungi (AMF) strain

*Rhizophagus irregularis* CD1, which is widely distributed throughout the world^26^, was used in the experiment. This fungi species was obtained from professor Zhaohui Chu’s laboratory and was cultured in a double culture system with a Ri-T-DNA carrot root^27,28^. A large number of hyphae and spores were collected on Ms medium without sucrose, and then the mycelium suspension was prepared. Five milliliters of spore suspension of approximately 2,000 spores was inoculated around the seed during sowing. The symbiosis between *R. irregularis* and upland cotton Lumian No. 1 was tested after 60 days.

### Plant

As one of the best cultivated varieties in China, Lumian No. 1 has many advantageous traits, such as a compact size, small main stem and branch angles, dark green hypertrophic leaves with deep wrinkles, and medium-sized cotton bolls. Lumian No. 1 also has many advantageous characteristics, such as a strong boll-forming tendency, early maturity, high yield, wide adaptability, strong resistance, and multiple flowers before frost. Nevertheless, lint percentage and single boll weight were 35%~37% and 5 g, respectively. Lumian No.1 is most suitable for planting in the middle and lower reaches of the Yellow River and greatly improves cotton production, promoting the development of cotton production and economic benefits in China.

### Experimental design cultivation management

A 2-year field experiment was conducted in a research field of Shandong Agricultural University, Tai’an, Shandong, China. For each year, the experiment was laid out in a factorial arrangement based on a randomized complete block design with noninoculated or inoculated *Rhizophagus irregularis* CD1 (Fig. S1). The experiment aimed to assess the effects of different inoculants under conditions of reduced fertilization; therefore, it included two treatments: (1) inoculated treatment, three replication plots; (2) noninoculated treatment, three replication plots. The acreage of each replication plot in the treatment was 22.5 m^2^ (15 m × 1.5 m). The planting row spacing and plant spacing were 0.8 m and 0.3 m, respectively.

Plastic sheeting was used to cover the young plants starting at 4 days after sowing. Once the emergence of seedlings was observed, the plastic sheeting was removed. Final singling was performed once the cotton seedlings had2–3 true leaves. Leaf branches and redundant sprouts were cut at the appropriate time. Timely control of aphids and cotton boll worms was performed during the whole growth period.

### Qualitative analyses of plant growth

The two cotyledons of the cotton were excavated when they turned from yellow to green and were spread out for germination. When 50% of the cotton seedlings in the whole field reached the standard of emergence, the date was recorded as the emergence period. The distance between the junction of the root and stem or the ground and the top of the main stem of the cotton seedlings was recorded as the seedling height, expressed in cm. The distance from the cotyledon node of the cotton seedling to the top of the main stem was recorded as the plant height, expressed in cm. The distance from the cotyledonary node to the first boll node was recorded as the first fruit branch height, expressed in cm. The average individual fruit branch number indicated the total number of fruit branches on August 15th. The total numbers of boll nodes on each plant boll branch were recorded as the whole boll nodes.

The net photosynthesis values were measured by a CIRAS-II (UK) portable photosynthesis measurement system from the first functional leaf, the fourth leaf from the top of the main stem. The distance from the leaf tip to the red point of the leaf base is the length of the functional leaf. The width of the functional leaf was measured through the red point of the leaf base and perpendicular to the vertical axis of the length. Then, the ratio of width/length (Rwl) was calculated. The seedling was considered a strong seedling if the leaf width was greater than the length; otherwise, the seedling was considered a weak seedling. The measurement method is shown in Fig. S2.

### Calculation of seed cotton yield

In this study, the spacing in the rows and the row spacing of the treatment plots was0.3 m and 0.8 m respectively. This spacing allowed the calculation of the number of plants per hectare. The computational formula is number of plants per hectare = 10000/(spacing in the rows × row spacing).

We counted the boll number and plant number in all the inoculated and noninoculated plots, respectively, and the boll number was divided by the plant number. Then, we obtained the mature boll number per plant. We also gathered all fibers in normally cracked cotton bolls and weighed them. Then, we obtained the single boll weight by dividing the weight by the quantity. The following formula was used to calculate the theoretical seed cotton yield.

$${\rm{Seed}}\,{\rm{cotton}}\,{\rm{yield}}\,({\rm{kg}}/{{\rm{hm}}}^{2})=\frac{{\rm{Plants}}\,{\rm{number}}\,{\rm{per}}\,{\rm{hectare}}\times {\rm{The}}\,{\rm{mature}}\,{\rm{boll}}\,{\rm{number}}\,{\rm{per}}\,{\rm{plants}}\times {\rm{Single}}\,{\rm{boll}}\,{\rm{weight}}\,({\rm{g}})}{1000}$$

### Quality analysis of cotton fiber

The mature fiber samples were collected and sent to the Cotton Research Institute at the Chinese Academy of Agricultural Sciences for standard testing.

The HVI system measures the average length of the upper half of the fiber in hundredths of an inch, converting the length to thirty seconds an inch. The length uniformity index is the ratio of the average length in percentage to the upper half average length. The fiber strength is measured in grams per tex and the force to break a bundle of fibers is expressed in grams. The official cotton standard for micronaire or fiber fineness and maturity is described as providing micronaire readings for air flow instrument testing to measure this quality in accordance with established procedures. The ratio of the length at which the stretched fibers broke and the original length is called the elongation percentage.

### Determination of phosphorus concentration in plants

The aboveground and belowground plant parts at 72 dpi under field conditions were used to measure phosphorus concentration. For total phosphorus concentration, the material was dried at 80 °C and then ground in a mortar. The detailed determination method can be reviewed in Zubek *et al*.^29^. Inorganic phosphorus concentration was measured according to the method reported by Shu, Francis^30^.

### Expression detection of phosphate transporter family genes

The CDS sequence of the cotton phosphorus transporter family genes was downloaded from the public CottonGen or the NCBI database according to the GeneBank ID. Supplemental Table S1 listed the IDs of phosphorus transporter family genes and primers.

According to the manufacturer’s instructions (T9424, Sigma-Aldrich, USA), total RNA was isolated from 50 mg plant tissues with TRI reagents. For first-strand cDNA synthesis, the *EasyScript*^®^ One-Step gDNA Removal and cDNA Synthesis SuperMix (TRAN, Beijing, CN) was used. RT-PCR was performed using an ABI 9902 (Applied Biosystems, Veriti® 96-Well Thermal Cycler 9902). The PCR program was as follows: 95 °C with 6 min, 35 cycles of 95 °C with 30 s, 60 °C with 30 s, 72 °C with 30 s, and a final step of 72 °C with 2 min. RT-qPCR was performed with UltraSYBR Mixture (Low ROX) using an Applied Biosystems 7500 System (Thermo Fisher, USA). The PCR program was as follows: 95 °C with 10 min, 40 cycles of 95 °C with 15 s and 60 °C with 1 min. Melting curve analysis (95 °C with 15 s, 60 °C with 1 min, 95 °C with 15 s and 60 °C with 15 s) was used for determination of the amplified PCR products specificity. The results were standardized with *GhUB7* (DQ116411) of *G. hirsutum* as internal control^31^. Quantitative PCR analysis was repeated at least twice in triplicate for each gene. The relative expression level was determined by 2^−∆∆Ct^ analysis.

### Statistical analysis

The Solutions Statistical Package in Social Sciences software version 19.0 was carried out for statistical analyses. To analyze data, One-way ANOVA was used, with “treatment” as the factor. To test the differences among treatments, Tukey’s honestly significant differences (HSD) with cutoff significance at P < 0.05 or P < 0.01 was performed. The detailed analysis method can be reviewed in Schüßler *et al*.^1^.

## Results

### Effect of AMF on the growth of Lumian No. 1

The seeding ratios of +AMF and −AMF are shown in Table 1 and were counted at 5 day and 7 day after sowing. The ratio was significantly different between the +AMF plots and −AMF plots, and the presence of +AMF plots increased the rate of emergence within a certain time frame compared to the −AMF plots (Table 1).

**Table 1:** Statistics on seedling emergence rates at 5 and 7 days after sowing.

Plot		Survey date	Seeding number	Sowing number	Seeding Ratio	Survey date	Seeding number	Sowing number	Seeding Ratio
* +AMF	Plot 1	5 days after sowing	5	50	0.100	7 days after sowing	46	50	0.919
	Plot 2		8	50	0.160		47	50	0.930
	Plot 3		3	50	0.060		42	50	0.849
−AMF	Plot 1		1	50	0.020		27	50	0.535
	Plot 2		0	50	0.000		44	50	0.872
	Plot 3		0	50	0.000		34	50	0.686

*show a significant difference at P < 0.05 of statistical analysis between treatments.

**Figure 1:**
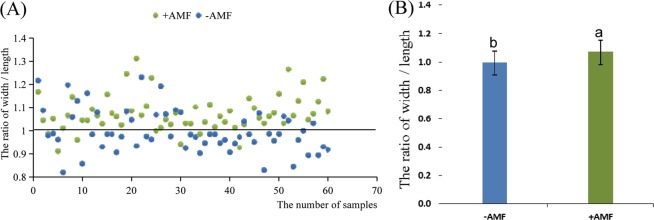
The width/length ratio of the functional leaf. (**A**) Scatter plot of the distribution of strong and weak functional leaves; (**B**) Statistical analysis of the difference between strong and weak functional leaves. If the value of width/length >1, the cotton seedlings will grow rapidly. If the value of width/length <1, the cotton seedlings will grow slowly. The proportion of strong seedings with (+AMF) and without (−AMF) AMF are 90% and 35% respectively. The means ± standard error values are shown in the figure. Bars labeled with a different letter at the top of each parameter show a significant difference at P < 0.05 by one-way ANOVA.

**Figure 2:**
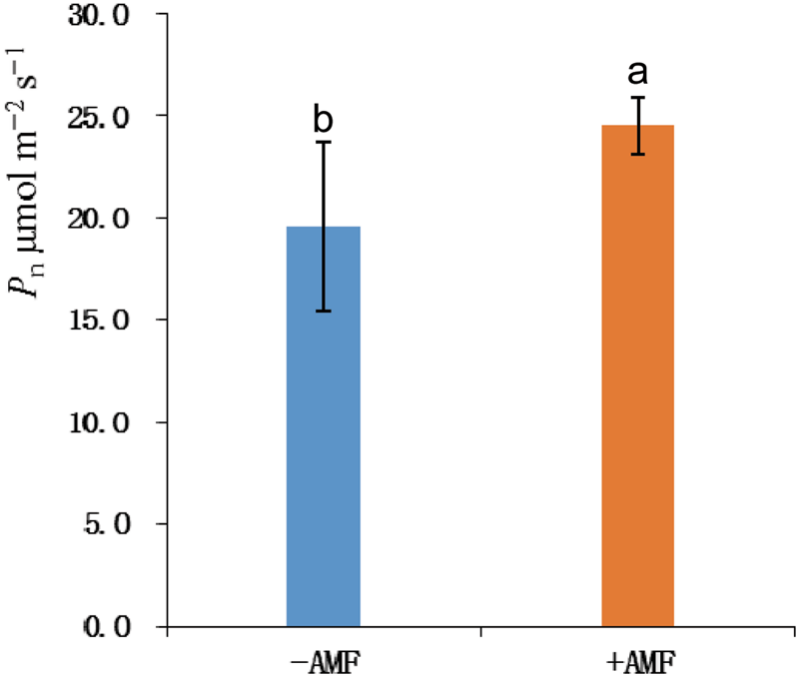
Net photosynthetic rate (*P*_n_) of upland cotton (Lumian No. 1) seedlings. The means ± standard error values are shown in the figure. Bars labeled with a different letter at the top of each parameter show a significant difference at P < 0.05 by statistical analysis of a single-factor experiment. The values were measured by CIRAS-II,UK portable photosynthesis measurement system. The unit of Net photosynthetic rate is μmol m^−2^ s^−1^.

The effect on seedling growth was significantly different between the +AMF and −AMF plots 40 days after sowing, and the functional leaf Rwl (ratio of width/length) was further analyzed, which is related to photosynthesis and seedling growth intensity^32^. Seedlings were classified as relative strong seedlings if the leaf width was greater than the leaf length (Rwl >1), while seedlings were classified as relative weak seedlings if the leaf length was greater than the leaf width (Rwl <1). In Fig. 1A, most of the green points (Rwl of the +AMF plots) are above the line with a value of 1, and most of the blue points (Rwl of the −AMF plots) are below line. One-way ANOVA indicated that AMF significantly affected the strength of the cotton seedlings, as shown in Fig. 1B. Photosynthesis intensity is an important index for measuring plant seedling growth ability, and the plants with strong photosynthesis will grow faster and accumulate more biomass. The detection of net photosynthesis in functional leaves showed that photosynthesis in +AMF plots were significantly stronger than that in−AMF plots, based on statistical analysis by one-way factor ANOVA (Fig. 2). The fourth leaf from the top of the main stem was the functional leaf. Therefore, AMF can improve the photosynthesis of plants.

**Figure 3:**
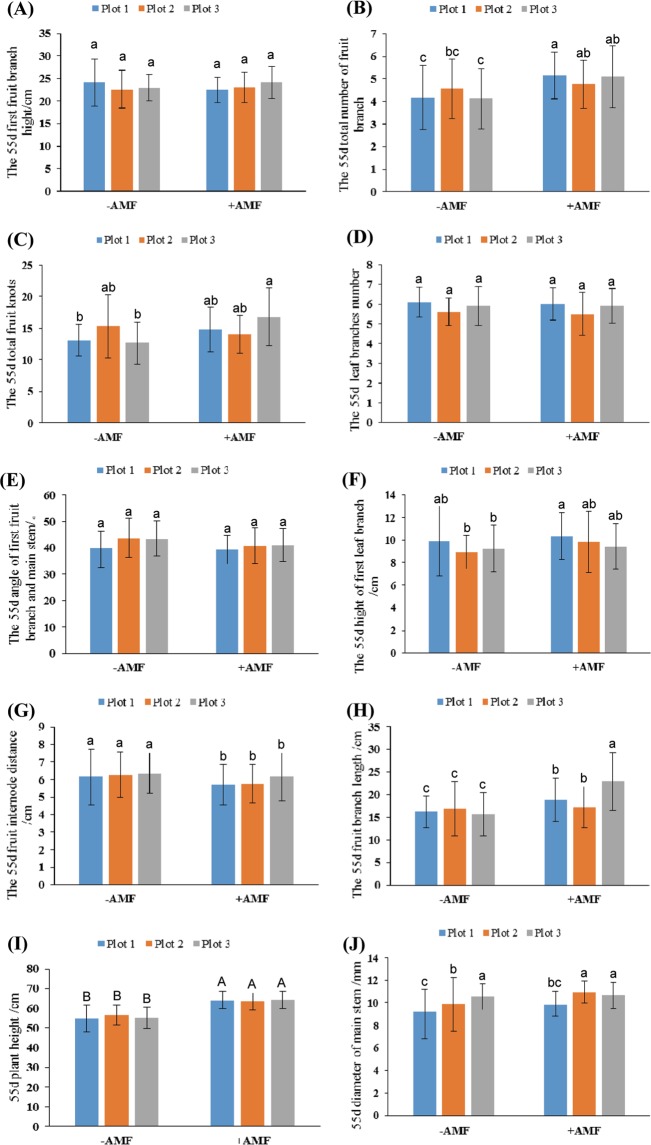
Plant growth traits at 55-days after sowing. (**A**) 55-day first fruit branch; (**B**) 55-day total number of fruit branches; (**C**) 55-day total boll nodes; (**D**) 55-day number of leaf branches; (**E**) 55-day angle of the first fruit branch and the main stem; (**F**) 55-day height of the first leaf branch; (**G**) 55-day fruit internode distance; (**H**) 55-day fruit branch length; (**I**) 55-day plant height; (**J**) 55-day diameter of the main stem. The means ± standard error values are shown in the figure. Bars labeled with a different letter at the top of each parameter show a significant difference at P < 0.05 by statistical analysis of a single-factor experiment. Bars labeled with a different capital letter at the top of each parameter show a significant difference at P < 0.01 by statistical analysis of single-factor experiment.

Fifty-five days after sowing is a very important time for AMF colonization, so agricultural traits were surveyed at this time point. More than 80% of cotton roots 25 cm below the soil surface had established mycorrhiza at the point of inoculation within 36 days^22^, and mycorrhiza will secondarily spread in 10–13 days. The investigation revealed that, compared with the −AMF plots, +AMF plots had higher plant height (Fig. 3I), a slightly higher first leaf branch (Fig. 3F), no difference in the height of the first fruit branch (Fig. 3A) or the leaf branch number (Fig. 3D), much longer length of the fruit branch (Fig. 3H), lower fruit internodes distance (Fig. 3G), slight roughness at the main stem (Fig. 3J), and more fruit branches (Fig. 3B) and total boll nodes (Fig. 3C), and the same angle between the first fruit branch and the main stem (Fig. 3E). The height of the plant, fruit branch length and number, and the total number of fruit branches of the +AMF plots are significantly higher than those of the −AMF plots. Therefore, we can conclude that AMF obviously improved the vegetative growth ability of cotton.

**Figure 4:**
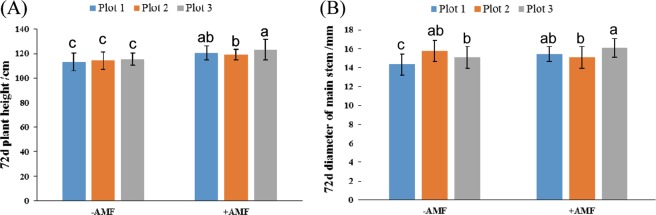
Plant growth traits at 72 days after sowing. (**A**) The plant height at 72 days after sowing; (**B**) the diameter of the main stem at 72 days after sowing. The means ± standard error values are shown in the figure. Bars labeled with a different letter at the top of each parameter show a significant difference at P < 0.05 by one-way factorial ANOVA.

Day 72 after sowing is an important period in cotton cultivation management. We controlled the height and fruit branches of the plants by removing the top of the cotton. At this point, as shown in Fig. 4, the height of the cotton obviously increased, and the diameter of the cotton became thicker in the +AMF plots compared with that in the −AMF plots.

**Figure 5:**
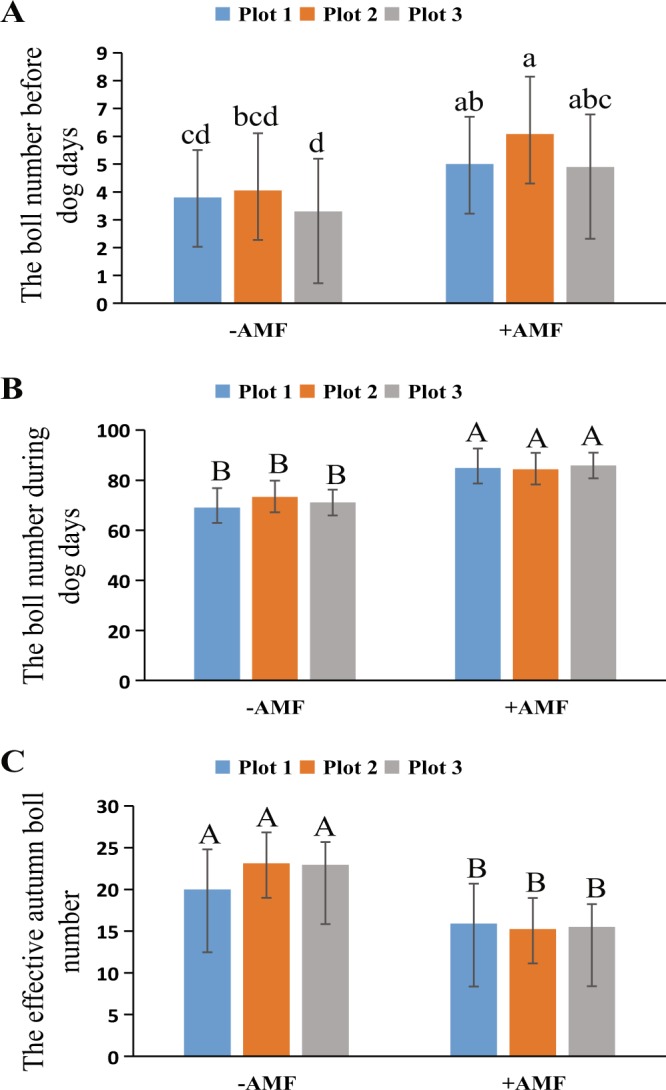
The boll number before late summer and during late summer and the effective autumn boll number. (**A**) The boll number before late summer; (**B**) the boll number during late summer; (**C**) the effective autumn boll number. The means ± standard error values are shown in the figure. Bars labeled with a different letter at the top of each parameter show a significant difference at P < 0.05 by statistical analysis of a single-factor experiment. Bars labeled with a different capital letter at the top of each parameter show a significant difference at P < 0.01 by statistical analysis of a single-factor experiment.

The bolls before late summer and during late summer are important components of cotton yield. The obvious increase in the quantity of the bolls before and during late summer can effectively improve the yield and quality of cotton fiber. The research shows an obvious increase in the quantity of bolls before and during late summer in +AMF plots compared with those in −AMF plots, as seen in Fig. 5A,B. The number of effective autumn bolls and the normal cracking bolls grown during autumn, were significantly increased in the +AMF plots compared with those in the −AMF plots (Fig. 5C). Thus, we can conclude that AMF has a strong effect on the reproductive growth of cotton.

**Figure 6:**
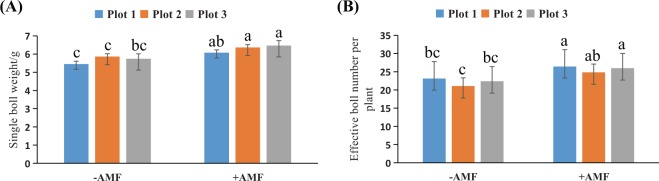
The boll traits in the harvest period. (**A**) The single-boll weight; (**B**) The effective boll number per plant. The means ± standard error values are shown in the figure. Bars labeled with a different letter at the top of each parameter show a significant difference at P < 0.05 by statistical analysis of a single-factor experiment.

Single boll weight and effective boll number per plant are important components of production. The results show that mycorrhiza-based inoculation significantly (ANOVA, P < 0.05) increased the mean single boll weight and effective boll number per plant (Fig. 6). Therefore, the total unginned cotton mean yield (theoretical value of formula calculation) of fiber increase was significant between inoculated (6759.3 ± 60.7 kg/hm^2^) and noninoculated control fields (5258.7 ± 30.0 kg/hm^2^) (Table 2). At straw cutting, all cotton plants in both inoculated and noninoculated plots showed evidence of mycorrhizal colonization.

**Table 2:** Statistical analysis of cotton yield.

Single boll weight		Boll number per plant		Plant number per hectare	Seed cotton yield, kg/hm^2^		Yield increase ratio, %
−AMF	+AMF	−AMF	+AMF		−AMF	+AMF	
5.46 ± 0.15	6.08 ± 0.29 *	23.13 ± 4.66	26.41 ± 3.16	41667	5258.7 ± 30.0	6759.3 ± 60.7	28.54
5.86 ± 0.16	6.36 ± 0.44 *	21.06 ± 2.26	24.84 ± 3.26				
5.74 ± 0.27	6.47 ± 0.62 *	22.39 ± 4.03	25.99 ± 3.27				

*show a significant difference at P < 0.05 of statistical analysis between treatments.

### Production and quality of cotton fiber

Statistical analysis showed a highly significant increase in yield (ANOVA, P < 0.05) for inoculated plots (6759.3 ± 60.7 kg/hm^2^) compared with that of the noninoculated controls (5258.7 ± 30.0 kg/hm^2^). The increase percentage was 28.54% (Table 2). At boll opening, all cotton plants in both inoculated and noninoculated plots showed evidence of mycorrhizal colonization, which was not surprising, as AMF occurs naturally in field soils. Cotton is an AMF-dependent crop^32^. However, current agricultural practices can reduce the diversity and abundance of AMF populations in the soil and increase the amount of time required to establish functional mycorrhiza^33^.

**Figure 7:**
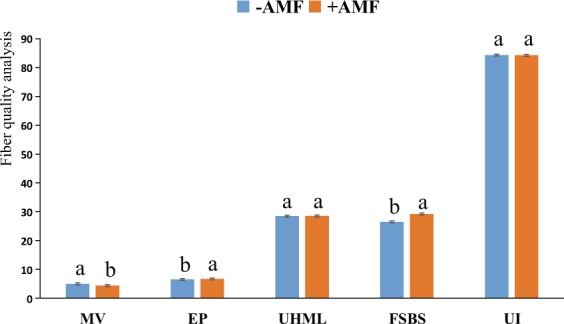
Fiber quality analysis. MV, micronaire value; EP, elongation percentage; AL, average length; FSBS, fiber specific breaking strength; UI, uniformity index; UHML, upper-half mean length. The means ± standard error values are shown in the figure. Bars labeled with a different letter at the top of each parameter show a significant difference at P < 0.05 by statistical analysis of a single-factor experiment. Each parameter was analyzed independently.

The elongation percentage (EP) is usually between 3% and 7%. In this study, the EP in the +AMF pots (6.6833%) was obviously better than that in the −AMF plots (6.4833%) (Fig. 7 and Table 3). A higher EP value is preferable, as it indicates that the fiber elasticity of the +AMF plots is better than that in the −AMF plots.

**Table 3:** Statistical analysis data on cotton fiber quality.

Items	Abbreviation			Fiber quality analysis	
		Plot	Mean	Standard errors	P < 0.05
Micronaire value	**MV**	−AMF	4.9667	0.2503	a
		+AMF	4.3800	0.1225	b
Elongation percentage, %	**EP**	−AMF	6.4833	0.0408	b
		+AMF	6.6833	0.0408	a
Upper half mean length, mm	**UHML**	−AMF	28.4667	0.4131	a
		+AMF	28.5167	0.6735	a
Fiber specific breaking strength (cN•tex^−1^)	**FSBS**	−AMF	26.4667	0.6314	b
		+AMF	29.2000	1.2083	a
Uniformity index, %	**UI**	−AMF	84.3667	0.9893	a
		+AMF	84.3000	0.6986	a

According to the classification rule, the fiber specific breaking strength (FSBS) of the +AMF plots (29.2) is considered strong (29.0~30.9), and the FSBS of the −AMF plots is considered moderate (26.0~28.9). The data are shown in Fig. 7 and Table 3.

Micronaire values (MV) are classified as A level (3.7~4.2 cN•tex^−1^, the best level), B level (B1 level, 3.5~3.6 cN•tex^−1^and B2 level 4.3~4.9 cN•tex^−1^) and C level (C1 level,<3.4 cN•tex^−1^and C2 level > 5.0 cN•tex^−1^). The MV of the +AMF plots (4.38 cN•tex^−1^), at theB2 level, was higher than the MV of the −AMF plots (4.9667 cN•tex^−1^), which was at theB2 and C level. The results indicate that the maturity of the +AMF plots was better than that of the −AMF plots. There was no significant difference in the upper half mean length (UHML, −AMF 28.4667, +AMF 28.5167) or the uniformity index (UI, −AMF 84.3667%, +AMF 83.3%) between the −AMF and +AMF treatments (Fig. 7 and Table 3). In conclusion, the inoculation of AMF improved the maturity of the cotton fiber and did not alter the decisive traits of the genetic material”.

### Phosphorus concentrations in roots, stems and leaves

**Figure 8:**
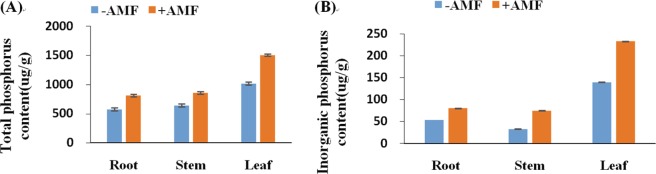
Phosphorus concentrations of plants in −AMF and +AMF plots. (**A**) The total phosphorus concentrations in −AMF and +AMF plots; (**B**) the inorganic phosphorus concentrations in −AMF and +AMF plots. The means ± standard error values are shown in the figure. Bars labeled with a different letter at the top of each parameter show a significant difference at P < 0.05 by statistical analysis of a single-factor experiment. Each parameter was analyzed independently.

The concentrations of P in Lumian No. 1 plant mass were influenced by AMF. In the case of Lumian No. 1, the plant was the most effective in the enhancement of P in root, stem and leaf concentrations. Higher concentrations of P in the roots, stems and leaves of Lumian No. 1 plants were found after *Rhizophagus irregularis* CD1 inoculation in comparison to those in the control (Fig. 8). Although inorganic phosphorus or total phosphorus concentration increased, the ratio of increase was different. The increase ratio of inorganic phosphorus in stems was the highest, followed by that of the leaves and roots. However, for total phosphorus, the highest concentration was in the leaves, followed by that in the roots and stems, as shown in Fig. 8A,B, respectively.

### The expression pattern analysis of phosphate transporters after mycorrhizal formation

**Figure 9:**
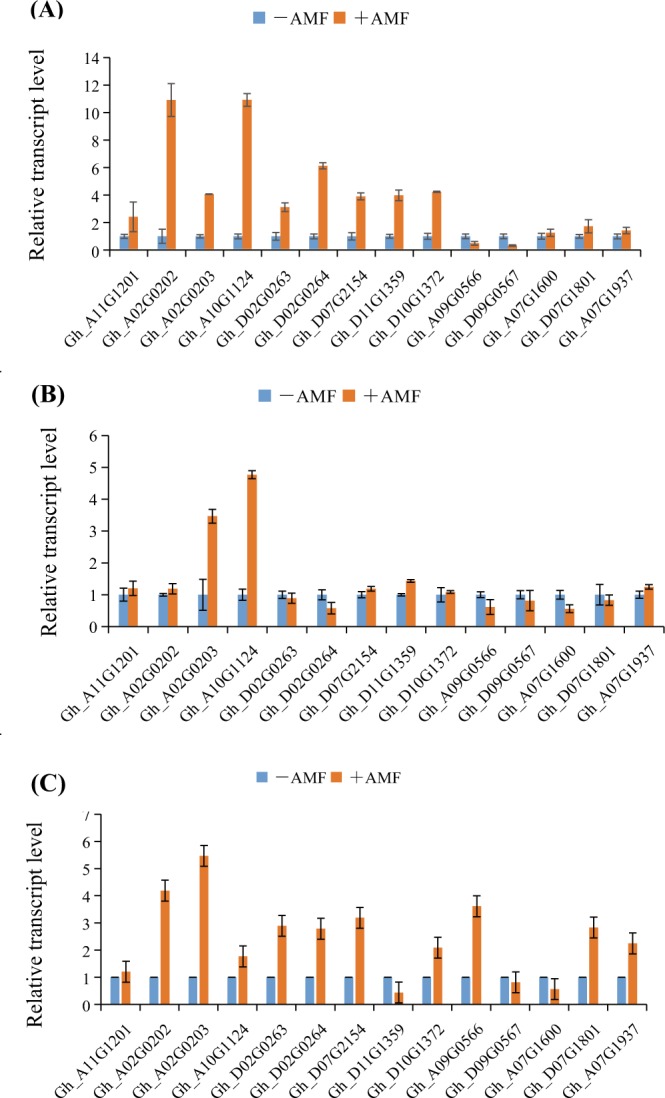
Expression patterns of phosphate transporters in leaves, stems and roots. (**A**) RT-qPCR analysis of phosphate transporter genes in leaves. (**B**) RT-qPCR analysis of phosphate transporter genes in stems. (**C**) RT-qPCR analysis of phosphate transporter genes in roots.

Most of the phosphate transporter family genes were upregulated in leaves, stems and roots. However, there were differences in the rates of upregulation in different tissues. Many more phosphate transporter family genes were activated and upregulated in leaves and roots than in stems, as shown in Fig. 9. This finding suggested that the physiological and biochemical reactions in leaves and roots are made more vigorous and complex through the symbiosis between cotton and AMF. P participates in more biochemical pathways, and the increase in P content causes the physiological metabolism of the plant to increase rapidly, which leads to the increase in the expression of P transporter family genes. In general, the expression of *Gh_A02G0203*, which may be an important gene for P transportation, was obviously increased in roots, stems and leaves.

## Discussion

Cotton is an AMF-dependent crop, so AMF inoculation is very important for cotton production^32^. The diversity of AMF reduction and loss of complementary benefits or well-performing plant-fungus combinations may be caused by human activities^34^. Adding AMF as inoculum can reduce this restrictions and improve plant growth^35^, as seen in the present study on cotton growth and in a study on tomato growth^4^. AMF population abundance and diversity in the farmland soil can be reduced by the current agricultural activities, increasing the amount of time required to establish functional mycorrhiza^33^. Previous studies have indicated that the important effect of inoculation of mycorrhizal fungi on P uptake^3,36^ and crop growth^1–5^, depended on early colonization. The introduction of vibrant AMF symbiotic microbes may help establish mycorrhiza earlier than the indigenous AMF populations would become established. Then, plant nutrition and growth benefit in the early growth stage. The inoculation propagules and quality are the main factors affecting functional AMF establishment^37,38^.

Inoculation of Lumian No.1 plants on a real cotton farm with the *Rhizophagus irregularis* CD1 mycorrhizal strain alone generated three main impacts, according to the research results: (i) increases in plant growth and in the P concentrations in the roots, stem and leaves, (ii) an increase in the seedcotton yield and (iii) improvements in the maturity of the cotton fiber.

### Phosphorus acquisition and regulation in AMF-based upland cotton

Several AMF studies have discovered that the intensity of root colonization was obviously affected by the host plant species^39,40^. Therefore, we used the cotton variety Lumian No.1 that had the strongest symbiosis with *R. irregularis* CD1. Delavaux *et al*.^3^ found that the increased soil P availability had a significant negative effect on AMF inoculation in farmland. The plant may directly obtains P from the soil, instead of exchanging carbon with the mycorrhiza-derived P. This effect has been reported in other studies^41–44^.

In this study, we found that, with AMF inoculation, the P concentrations in the roots, stems and leaves of cotton were increased. Experimental factors and their interactions significantly affected the P concentrations and contents in shoot^36^. The growth and P content of the invasive species *R. laciniata* and *S. gigantean* were enhanced by AMF^2^. The accumulation of P in the leaves of the plants inoculated with both fungi was higher than that of the plants inoculated only with bacteria and the control^5^. Phosphorus also inhibits AMF colonization in roots by inhibiting the expression of plant symbiotic genes, especially genes encoding carotenoids and lactones biosynthesis enzymes, as well as symbiotic related phosphorus transporters^45^. Therefore, lower levels of P may regulate the expression of P transporter family genes and then increase the trading ratio and the AMF inoculation speed. Therefore, this study is consistent with our research results.

Phosphate transporters are crucial gene family which play important role in phosphorus acquisition and regulation. Total genome-wide 14 gene members were inspected on expression pattern in cotton response to AMF inoculation. Both specific up-regulation and down-regultaion modes occured among the phosphate transporter family members, which suggested the positive and negative functional roles respectively in AMF-based cotton.

### Effect of AMF on the vegetative and reproductive growth of Lumian No. 1

The cotton growth was enhanced in our study in terms of the rate of emergence, strength of the cotton seedlings, height of the plant, fruit branch length and number, and the total number of fruit branches. The growth effect of AMF focus on the fruit branch related traits in AMF-inoculated cotton plants could be due to enhanced P uptake and/or transportation for fruit related growth/development and photosynthates. Previous findings indicated that AMF directly affect the early stages of *R. laciniata* and *S. gigantea* growth^2^. The growth and viability of tropical tree seedlings were improved with AMF inoculation, but the efficiency of the treatment may depend on plant and AMF genotype^1^. With AMF inoculation, the soil properties, aboveground and underground biodiversity, tree/shrub seedling survival, and establishment of moisture and nutrient stressed soils were significantly improved^46^.

AMF colonization promoted plant growth and reproductive growth. The maturity of the cotton boll was accelerated, and the time at maturity was prolonged. Subsequently, the fiber maturity grade was high. The inoculation with AMF only improved the maturity of the fibers and did not alter the decisive traits of the genetic material, as shown in the present research.

### Production of cotton fiber in upland cotton with AMF inoculation

Statistical analysis proved a marked increase in crop yield (ANOVA, P < 0.05) for the inoculated plots (6759.3 ± 60.7 kg/hm^2^) compared with the noninoculated controls (5258.7 ± 30.0 kg/hm^2^). The increase percentage was 28.54%, as shown in the present study. The components of cotton yield are plant number per hectare, mature boll number per plant and single boll weight. AMF can affect flowering, which is driven by the complex biological response of nutrient and carbon demand in plant tissue, which can be affected by AMF^47^. Torelli *et al*.^48^ and Boldt *et al*.^47^ reported that the enhanced photosynthates and phytohormones, which modulated by arbuscular mycorrhizal fungi, led to higher flower and fruit numbers in AMF-inoculated plants. AMF colonization can improve the adaptability of host species by affecting reproductive function^49^. Inoculated with AMF, fruits of plants were larger and heavier than those of uninoculated ones^4^. Fruits of olive plants colonized by AMF were contained more oil than those of control plants^50^. Analogously, the weight of cucumber was increased by AMF inoculation. The mycorrhiza-based inoculation of potato increased the total yield, and the mean yield increase was highly significant (P < 0.01) between inoculated (42.2 ± 1.03 tons/hm^2^) and noninoculated fields (38.3 ± 1.03 tons/hm^2^)^20^. Nevertheless, some negative effects on production have been reported; flower production decrease was observed with AM fungi compared with the control treatments^51^ and growth reduction was found in mycorrhiza-mediated wheat growth^18,19,52–54^. AMF extent differences have been found in four wheat cultivars^55^, suggesting that wheat varieties are not only different in response to colonization of AMF but also differ in soil resources exploitation ability. However, no effects were reported in a previous study in cultivar cotton.

In this study, a single strain of one AMF species, *Rhizophagus irregularis* (synonym *G. irregulare*) CD1, which exhibits substantial flexibility and adapts to different environment was used. However, particularly in P-rich soils^41–44^ and under highly stressful conditions^5,56^, *Rhizophagus irregularis* CD1 may still be limited to specific agricultural conditions. In conclusion, in our current study, we showed that the use of biofertilizer (AMF) in farmland is benefit for sustainable agriculture with two reasons: (a) chemical fertilization reduction and (b) enhancement of the yield and cotton fiber quality.

## Conclusions

Our investigation included one common AMF species (*Rhizophagus irregularis*). We reported here for the first time on the growth, yield and fiber quality of upland cotton in response to AMF inoculation. Our studies indicate that the expression of the specific phosphate transporter family genes was enhanced, and the P concentration in the cotton biomass was also increased (13.65%~43.27%) by AMF. The photosynthesis rate, plant growth, boll number per plant and maturity of fiber were increased through the symbiosis between cotton and AMF. Statistical analysis showed a highly significant increase in yield (ANOVA, P < 0.05) for inoculated plots (6759.3 ± 60.7 kg/hm^2^) compared with noninoculated controls (5258.7 ± 30.0 kg/hm^2^), with an increase percentage of 28.54%.

## Supplementary information

Supplementary Information.
