# Supplementary material for: Arbuscular mycorrhizal fungi (AMF) enhanced the growth, yield, fiber quality and phosphorus regulation in upland cotton (Gossypium hirsutum L.)
Source: Sci Rep. 2020 Feb 7;10:2084. doi: 10.1038/s41598-020-59180-3 (PMC7005850; doi:10.1038/s41598-020-59180-3)
Supplement: Supplementary file 1 — Supplementary Information. [file 41598_2020_59180_MOESM1_ESM.pdf]

Arbuscular mycorrhizal fungi (AMF) enhanced the growth, yield, fiber quality and phosphorus regulation in upland cotton (*Gossypium hirsutum* L.)

Xinpeng Gao[1, 2, a], Huihui Guo[1, a], Qiang Zhang[1], Haixia Guo[1], Li Zhang[1], Changyu Zhang[1], Zhongyuan Gou[1], Yan Liu[1], Junmei Wei[1], Aiyun Chen[1], Zhaohui Chu[1]* and Fanchang Zeng[1]*

[1] *State Key Laboratory of Crop Biology, Shandong Agricultural University, Tai'an 271018, P. R. China*

[2] *Novogene Bioinformatics Institute, Beijing 100083, P. R. China*

*Corresponding author E-mail

Zhaohui Chu: zchu@sdau.edu.cn, Fanchang Zeng: fczeng@sdau.edu.cn.

Mailing address: State Key Laboratory of Crop Biology, Shandong Agricultural University, Tai'an 271018, P. R. China

Tel: +86-538-8241828

[a] Equal contribution to this work

Supplementary Data:

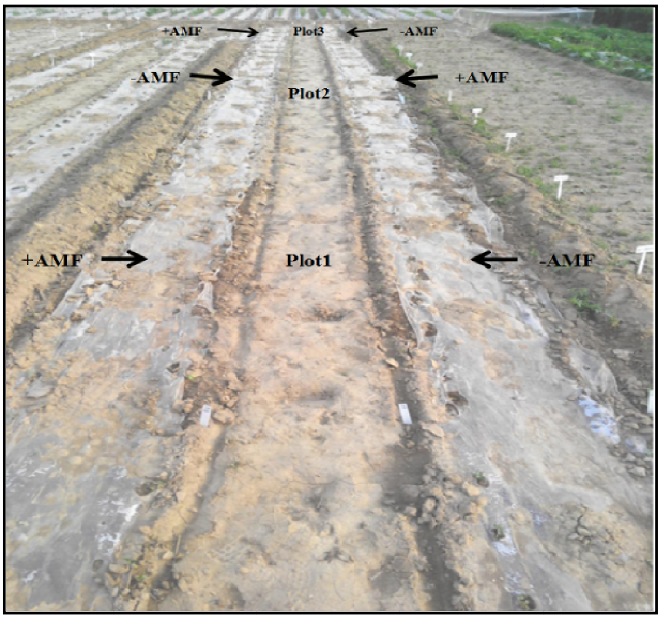

Fig. S1 The design of experimental plots in the field. +AMF indicate AMF was inoculated by *R. irregularis*, -AMF none.

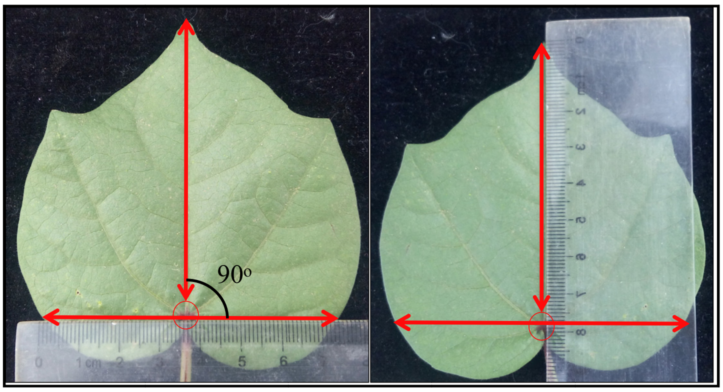

Fig. S2 Method for measuring leaf width/length. The fourth leaf from the top of the main stem was the functional leaf. The distance from the leaf tip to the red point of the leaf base is the length of the functional leaf. The width of the functional leaf is measured through the red point of the leaf base, perpendicular to the vertical length axis. The seedling was considered a relative strong seedling if the leaf width was greater than the length; otherwise, the seedling was considered a relative weak seedling.

Table S1 The sequences of phosphate transporter family genes primers in cotton

Primer name	Forward（sense) (5'-3')	Reverse (antisense) (5'-3')
Gh_D02G0263	TCAATGCGGAACAAGAGAAG	CCGCAGAGTGCAATAAGTGT
Gh_A02G0202	GCAGCTGCTGTTAATGGTGT	GGATAATCACCACCGATTCC
Gh_D02G0264	TTGGCGGATCATACTGATGT	CTTCTCTTGTTCCGCATTGA
Gh_A02G0203	GCAGCTGCTGTTAATGGTGT	GGATAATCACCACCGATTCC
Gh_A07G1937	CTGGGATGGGTTTCTTCACT	TTATCACCAAGCCAACCAAA
Gh_D07G2154	GGCTATCCCGTATGACCACT	GACGAATGTGGTGGCATTAG
Gh_A07G1600	TGGGAGTGATCAGCTTCTTG	TCTGTTGTCCACCCTGTGTT
Gh_D07G1801	ATCGAAAGGGAAATCACTGG	TCGTCCACCTTGTGTTTGAT
Gh_A09G0566	CTATTGCCTCTGGCTTGTCA	TAAGCAGGTGCCTTGTATGC
Gh_D09G0567	ATCCCGGCTGCTCTTACTTA	CTTTGCAACAAGGGCAGTAA
Gh_A10G1124	ATCGCTTATCGCACTTTGTG	ATTGAATTGTGAACCTGCCA
Gh_D10G1372	ATCGCTTATCGCACTTTGTG	ATTGAATTGTGAACCTGCCA
Gh_A11G1201	TGGTGCATTTGGATTCTTGT	GACTCCAATACCAGCAGGGT
Gh_D11G1359	ACGGTGTTCATGTTTGCACT	ACAAGAATCCAAATGCACCA
GhUB7	GAAGGCATTCCACCTGACCAAC	CTTGACCTTCTTCTTCTTGTGCTTG
